# Quercetin inhibits fibroblasts proliferation and reduces surgery-induced epidural fibrosis via the autophagy-mediated PI3K/Akt/mTOR pathway

**DOI:** 10.1080/21655979.2022.2062530

**Published:** 2022-04-12

**Authors:** Yile Cao, Hui Chen, Yu Sun, Zhehao Fan, Hong Cheng

**Affiliations:** a Department of Clinical Medicine, School of Medicine, Yangzhou University, Jiangsu, Yangzhou, China; b Department of Orthopedics, Clinical Medical College of Yangzhou University, Yangzhou, Jiangsu, China; c School of Medicine, Yangzhou University, Jiangsu, Yangzhou, China; d Yangzhou University Medical College, Jiangsu Key Laboratory of Experimental & Translational Non-coding RNA Research, China Institute of Translational Medicine, Yangzhou University, Jiangsu, Yangzhou, China

**Keywords:** Quercetin, autophagy, proliferation, apoptosis, epidural fibrosis (EF)

## Abstract

Epidural fibrosis (EF) is a serious complication when the patients suffer from operations of lumbar laminectomy. It is reported that quercetin plays a positive role in the prevention of various fibrotic diseases. However, the role of quercetin in the prevention of epidural fibrosis (EF) and its possible mechanism are unclear. Fibroblast proliferation is considered to be the main cause of epidural fibrosis.Autophagy is a lysosomal degradation pathway that is essential for survival, differentiation, development, and homeostasis.Although autophagy has been associated with fibrosis of different tissues, the effect of autophagy on epidural fibrosis is still unknown.The aim of this study was to investigate the function and mechanism of autophagy induced by quercetin, a polyphenol derived from plants. In vivo, the effect of quercetin on reducing epidural fibrosis was confirmed via histological staining and immunohistochemical analysis. The results showed that quercetin had significant suppressive effects on epidural fibrosis following laminectomy in rats.In vitro,, cell counting kit-8 (CCK-8), Western blot analysis, immunofluorescence and Edu staining, TUNEL staining and transmission electron microscopy were used to detect the effects of quercetin on the proliferation and apoptosis of fibroblasts and explore the possible signal transduction pathway. Results indicated that quercetin could induce autophagy and inhibit proliferation in fibroblasts. In conclusion, Quercetin could regulate fibroblast proliferation, apoptosis, migration and other biological behaviors through autophagy, thereby preventing epidural fibrosis. The specific corresponding pathway may be the PI3K/Akt/mTOR signaling pathway.

## Graphical abstract

**Figure:**
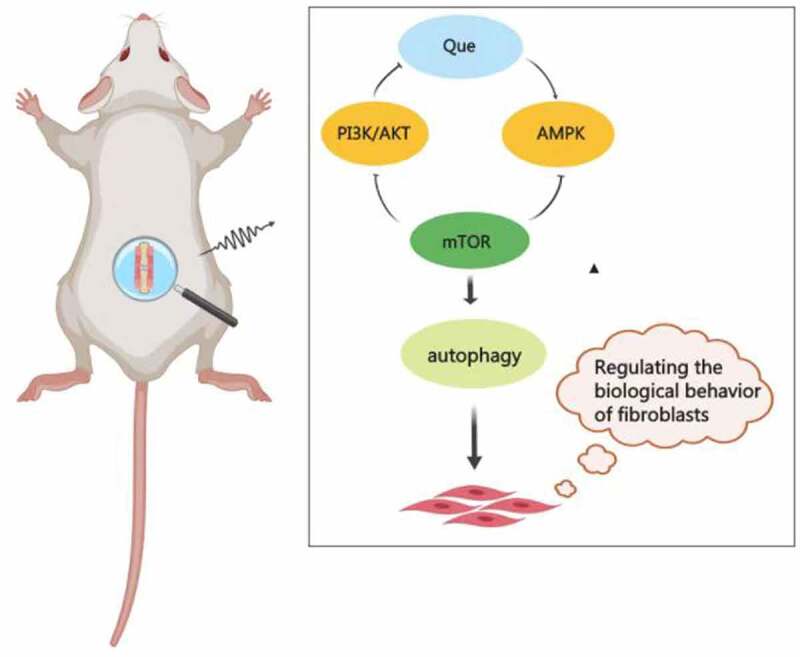

## Introduction

Operations involving the spinal canal can inevitably cause damage to the peridural structure, resulting in the formation of epidural fibrosis (EF) [1,2]. During patient recovery and wound repair, local fibroblasts are activated and migrated, leading to spinal canal stenosis and compression of the patient’s dural sac and nerve root, which may eventually lead to fibrosis and adhesion and give rise to pain. Therefore, the prevention of EF is of great significance to both surgeons and patients who require surgery [3,4]. Thus far, the pathogenesis of EF and scar adhesion has not been fully studied, however, most scholars believe that the activation, migration and excessive proliferation of fibroblasts in the surgical area are the main causes of the disease [5,6]. Therefore, we believe that adopting effective measures to prevent excessive proliferation of fibroblasts can serve as a reliable means in preventing EF.

Currently, in order to prevent and treat the occurrence of EF and postoperative pain, many methods have been adopted clinically, which mainly include: improved operation to reduce bleeding, drug treatment during and after operation, implantation of biomaterials, and rehabilitation exercise [7–9]. It is of great clinical significance to identify novel therapeutic and pharmacological targets in order to prevent EF with little side effects [6].

Autophagy is a very conservative biological process in cells [10]. It is believed that the main function of autophagy is to remove damaged cell structures, senescent organelles and various macromolecules that are no longer used in cells [11–13]. Autophagy, which plays an important role in growth and development, is closely connected with cell differentiation and the stress response [14].Previous studies indicated that autophagy could downregulate mTOR expression to inhibit cell proliferation [15,16].The mTOR protein is responsible for regulating cell cycle and cell proliferation [17].Previous studies have shown that PI3K/Akt/mTOR signaling pathway is widely involved in fibroblast proliferation [18].Blocking mTOR signaling pathway can inhibit cell proliferation and induce apoptosis. These evidences indicate that cellular autophagy cascade can regulate cell proliferation via PI3K/Akt/mTOR signaling pathway.

Quercetin (3ʹ,4ʹ,5,7-pentahydroxyflavone) can be widely found in foods, plants, and beverages [19]. Quercetin has been reported to be critical to numerous fibrotic diseases. In vivo, quercetin inhibits PI3K/Akt pathway, thereby reducing renal fibrosis and apoptosis in CRF rats [20].

However, whether quercetin can affect cell proliferation, apoptosis and autophagy of fibroblasts in EF has yet to be investigated. Does quercetin have a positive effect in reducing surgery-induced intraarticular fibrosis scar adhesion? What is the role of the PI3K/AKT/mTOR signaling pathway in quercetin-induced intraarticular fibrosis reduction? This study attempts to explore these problems and propose a new, effective and simple technique to treat intra-articular fibrosis.

## Materials and methods

### Rat grouping

All animals were duly cared for in accordance with experimental animal protection guidelines, and this study was approved by Yangzhou’s Animal Ethics Committee (license number: YIACUC-19-0023), all rats received scrupulous care .We researched 36 adult male SD rats (Yangzhou, China) weighing 280 ± 20 g who were then purchased from the laboratory animal center of Yangzhou University. The samples were arbitrarily separated so as to comprise three groups (12 rats per group): quercetin (100 mg/kg), quercetin (200 mg/kg) and the control group (saline) [21,22].

### Laminectomy modeling and intragastric administration of quercetin

Rat laminectomy models were built in line with previous studies [23,24]. Briefly, following satisfactory anesthesia by ketamine (100 mg/kg body weight) via intraperitoneal injection, the samples’ fur were shaved within L1 and L2, and the visible skin was disinfected using iodophor. Applying an incision on the midline skin and a detachment of the paravertebral muscles, the L1 vertebral plate was made visible. By detaching the spinous process and L1 vertebral plate using rongeur forceps, the dura mater was uncovered. According to this method, L1 laminectomy was performed in rats, after which the experimental model was obtained. Penicillin (50 mg/kg) was injected intramuscularly in each rat to prevent incision infection, once a day for three days.

From day 1 after laminectomy, the rats were gavaged with quercetin by intragastric administration in different doses (100 mg/kg and 200 mg/kg) or the control group (saline) once a day for 1 month. In this month, all rats were carefully cared for.

## Histological analysis

Anesthesia was administered on the rats prior to applying 4% paraformaldehyde as an intracardiac perfusion. Overall, the L1 spinal column, alongside epidural fibrous tissue and paravertebral muscles, were detached for soaking using a 10% formalin buffer, which was then softened and paraffin-embedded. Through the L1 vertebra, continuous 4-μm transverse incisions were carried out. Masson and hematoxylin-eosin (H&E) were used for dyeing. Employing a × 400 magnification on a light microscope (UVIGAS7001B, UK), the degree of epidural scar adhesion and epidural fibrosis was then determined.

### Immunohistochemistry

Immunohistochemical staining was performed using previously reported methods [25].The slices were cooled and rinsed in PBS 3 times, and a sufficient amount of H2O2 solution was dripped onto the slices and rinsed thoroughly. The prepared primary antibodies recognizing p-mTOR and LC3 were added to the slices and stored in a 4°C refrigerator wet box overnight. Then, it was incubated with anti-mouse IgG for 1.5 hours at room temperature, and antibody binding was detected using a DAB staining solution. Eventually, the samples were de-stained using hematoxylin for 3 minutes, after which the fibroblasts were determined through an optical microscope. The expression of p-mTOR and LC3 was indirectly evaluated by calculating the percentage of immunopositive cells using the ImageJ software.

### Fibroblast culture and treatment

According to our previous research, the primary fibroblast cell line was obtained [26]. Using Dulbecco’s modified Eagle’s medium (DMEM; Gibco, Grand Island, NY, USA), cultured cells were attained at 37°C and 5% CO_2_. The medium was supplemented with penicillin with a concentration of 100 U/ml, 15% fetal bovine serum (FBS; Gibco, Grand Island, NY, USA), and streptomycin with a concentration of 100 mg/ml (PS; Thermo, Rockford, IL, USA). The cells were cultured to 3–6 generations and those exponentially growing were used as the experimental materials. The fibroblasts were seeded in 96-well plates, 6-well plates or 10-cm culture dishes overnight. After the cell density reached 50 ~ 80%, 7.4-pH phosphate buffered saline (PBS) was used to wash the cells, which were then treated with Sigma-brand quercetin (St. Louis, MO, USA).

### Cell viability assay

Cell viability was measured using Cell Counting Kit-8 (CCK-8, Dojindo, Tokyo, Japan). Cells were seeded in 96-well plates in triplicate and treated separately with 0 to 80 μmol/L quercetin dilutions. In another group, cells were cultured in DMEM for 0, 12, 24, 36, 48, 60, and 72 hours, after which the cells were incubated with 10 μL of WST-8 (Dojindo Laboratories, Kumamoto, Japan) for 1 hour at 37°C. Cells positive for WST-8 staining were considered to be viable cells.

### EdU incorporation assay

Cell proliferation was measured using Cell-Light KFluor488 EdU Kit (Ribobio, Guangzhou, China). Fibroblasts with a density of 1 × 10^5^ were seeded into 6-well plates and incubated at 37°C for 24 hours. They were then treated with 20 μmol/L quercetin for 24 hours. Then, 50 μM EdU was added to each well for 2 hours. The cells were immobilized with 4% paraformaldehyde for 10 minutes and then infiltrated with 0.5% Triton X-100 for 15 minutes. The nuclei were stained with Hoechst 33,342 and were then observed under an inverted fluorescence microscope.

### TUNEL assay in fibroblasts

TUNEL assay was performed using previously reported methods [27].In order to assess human fibroblast apoptosis, TUNEL staining was conducted (KeyGEN, Nanjing, China). Trial procedures strictly complied with the directions given by the manufacturer. In order to perceive the apoptosis attributes, a fluorescence microscope was utilized. It was deemed apoptotic if the fibroblasts were TUNEL-stained. Through DAPI staining, the overall fibroblast count was determined.

### Western blot analysis

Using a radioimmunoprecipitation assay (RIPA) buffer (Beyotime, Shanghai, China), the total protein was acquired. In addition, a BCA Protein Assay Kit (Beyotime, Shanghai, China) was utilized in the determination of total protein concentration. Through a 5–12% sodium dodecyl sulfate- polyacrylamide gel electrophoresis (SDS-PAGE), 50-μg protein extracts were detached from being transmitted into membranes containing polyvinylidene difluoride (Millipore, Bedford, MA, USA). The membranes were blocked for two hours at room temperature using 5% skim milk before being nurtured using primary and secondary antibodies, in line with the provided directions. Eventually, the protein bands were noted through an improved chemiluminescence determination (ECL- Plus kit, Beyotime, Shanghai, China), which were then digitally taken (Bio-Rad ChemiDoc XRS+, California, America). Blotting with β-actin regulated the differences in protein loading. Using the densitometry of the unsaturated captures and an exclusion of the background density, immunoreactive bands were then counted (Image J, NIH, Bethesda, USA). The antibodies were listed in the following: PCNA, Cyclin D1, cleaved-PARP, α – SMA, collagenI, collagen III, LC3, PI3K, p-PI3K, AKT, p-AKT, MAPK, p-MAPK, mTOR and p-mTOR(dilution 1:1000,CST, USA), Bax (dilution 1:1000, Abcam, China), β-actin (dilution 1:2000, CST, USA) and secondary antibody(dilution 1:5000, CST, USA).

### Immunofluorescent staining

Cells were treated with 0 and 20 μmol/L quercetin, respectively, then fixed with 4% paraformaldehyde for 10 minutes. After washing, permeation was conducted for 15 minutes using 0.1% Triton X-100. Cells were incubated overnight at 4°C using anti-α-SMA (1:100) and anti-LC3 antibody (1:100). It was performed using an antibody made of anti-rabbit IgG H&L 1 hour after washing (Alexa Fluor 555) (1:200) (Abcam, Cambridge, MA, USA).

## Statistical analysis

Statistical Product and Service Solutions (SPSS) 22.0 (IBM, Armonk, NY, USA) was used in the analysis of the experimental data. All quantitative data were presented as mean ± standard deviation (SD). Percentage (%) was used to express the enumeration data. Continuous variables were analyzed using a chi-square test or Student’s t-test, depending on the distribution of the variables. Comparison between multiple groups was done using One-way ANOVA test followed by Post Hoc Test (Least Significant Difference). P values < 0.05 were considered statistically significant.

## Results

In the present study, we aimed to investigate whether quercetin could reduce intraarticular fibrosis scar adhesion by inhibiting fibroblast proliferation and promoting fibroblast apoptosis in vivo and in vitro studies. According to the results of pathological observation, local application of quercetin can reduce the degree of postoperative intra-articular adhesion.In vitro studies, Quercetin can inhibit the viability and reduce the number of fibroblasts through the regulation of proliferation, apoptosis, and migration.Mechanistically, we demonstrated that quercetin regulates fibroblast apoptosis and proliferation through autophagy, and mTOR mediated signaling pathway is involved.

### Macroscopic and histological evaluation of EF

**Figure 1.**
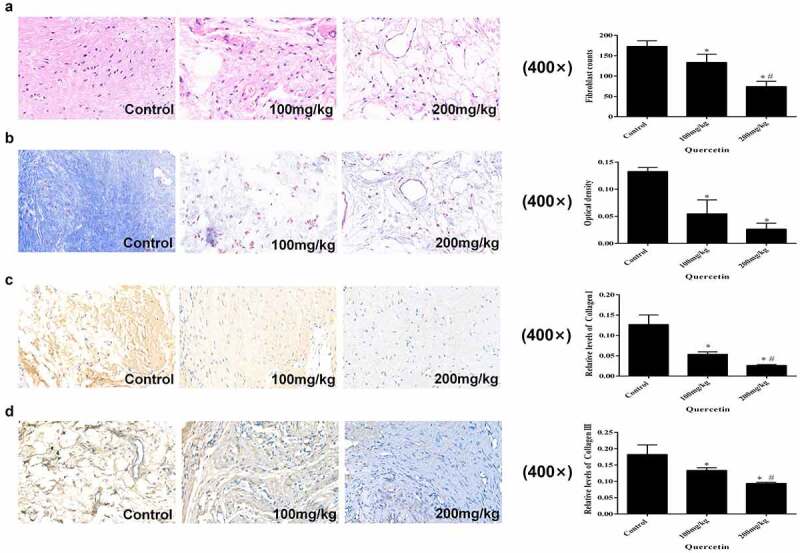
Macroscopic and histological evaluation of EF. (a) The count of fibroblasts in epidural fibrosis area decreased in a dose-dependent manner in images of H&E staining (magnification, ×400). (b) Representative images of Masson staining and the OD value of collagen (magnification, ×400).Quercetin inhibited epidural collagen synthesis in a dose-dependent manner.Intra articular collagen appeared blue. (c,d) Representative images of Masson staining and the relative levels of collagen I and collagen III (magnification, ×400). (*Compared with Control group, P < 0.05; #Compared with 100 mg/kg group, P < 0.05).

Cell counting of the HE stained sections demonstrated that as the concentration of quercetin increased, fibroblast counts decreased. This results indicated that intragastric administration of quercetin could reduce the number of fibroblasts in the operation area in a dose-dependent manner (*p* < 0.05, Figure 1(a)). Masson staining showed that the collagen content in the 200 mg/kg group was the lowest (*p* < 0.05, Figure 1(b)), decreasing collagen I and collagen III trends were related to the rise in drug concentration (*p* < 0.05, Figure 1(c-d)). These results indicated that the collagen content in the operation area decreased along with the increase in quercetin concentration.

### Quercetin induces autophagy in the surgical area

**Figure 2.**
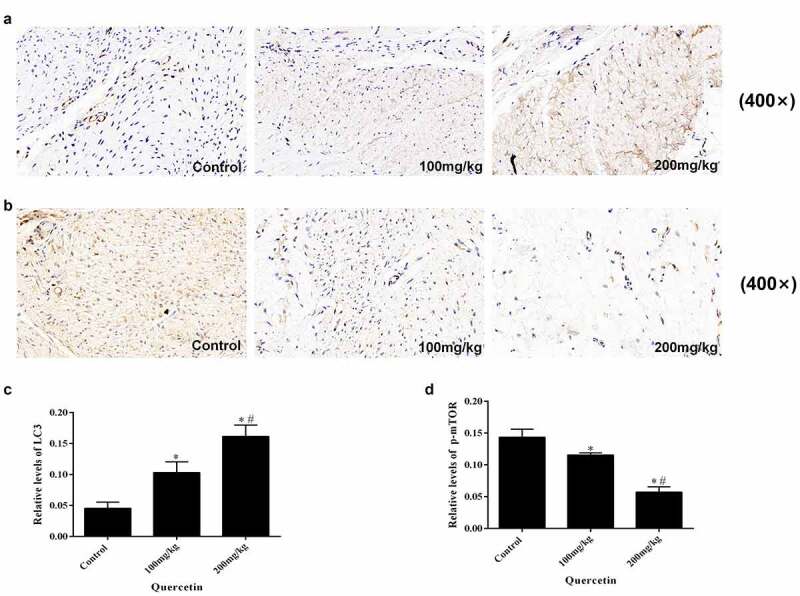
Immunohistochemical staining of LC3 and p-mTOR in epidural fibrosis tissues. (a, c) LC3 immunohistochemistry staining results showed that quercetin treatment reduced the number of LC3-expressing cells in a dose-dependent manner in rats(magnification, ×400). (b. d) p-mTOR immunohistochemistry staining results showed that quercetin treatment reduced the number of p-mTOR-expressing cells in a dose-dependent manner in rats(magnification, ×400). (*Compared with Control group, P < 0.05; #Compared with 100 mg/kg group, P < 0.05).

In order to explore the effect of quercetin on autophagy, the expression of p-mTOR and LC3 in epidural tissue sections were tested using immunohistochemistry. The results illustrated that the expression levels of LC3 gradually increased while the expression of p-mTOR decreased along with the concentration of quercetin, exhibiting a concentration-dependent manner (*p* < 0.05, Figure 2(a-d)). It can be concluded that quercetin can induce autophagy following epidural surgery, for which the mTOR mediated signaling pathway is involved.

### Quercetin affects the biological behaviors of fibroblasts such as apoptosis, proliferation and migration

**Figure 3.**
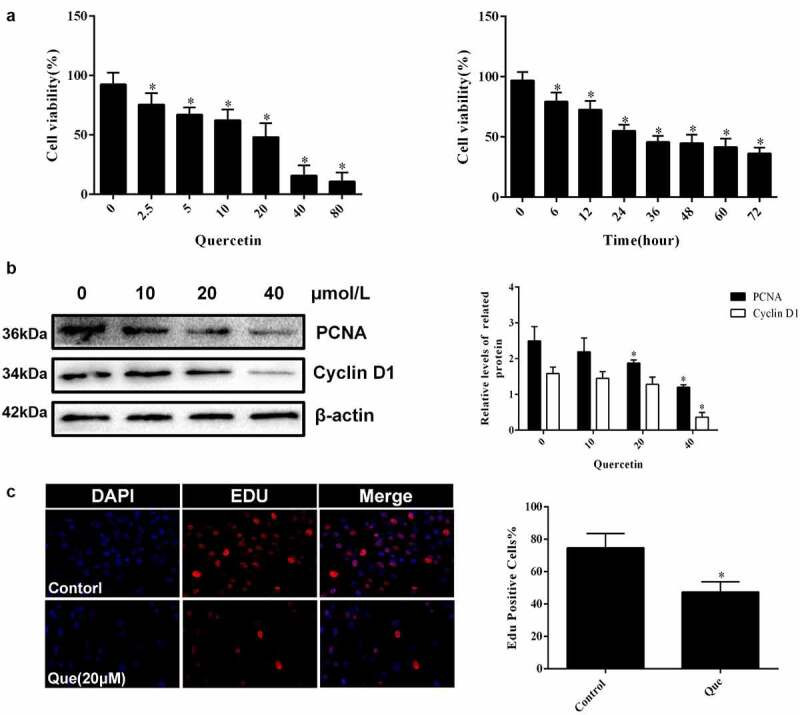
Quercetin inhibited fibroblast proliferation in vitro. (a) CCK-8 showed that the viability of fibroblasts decreased with the increase in quercetin concentration .(b) CCK-8 showed that the viability of fibroblasts was found to decrease with time in a time-dependent manner after the treatment with 20 μmol/L quercetin (c)Edu staining showed that the number of positive cells decreased significantly after the treatment with 20 μmol/L quercetin for 24 h . (d) The proteins levels of PCNA and Cyclin D1 decreased in a concentration dependent manner treated with quercetin .β-actin was used as a control.

In regard to the investigation of the specific effect of quercetin on fibroblasts, cytological experiments were carried out. CCK-8 showed that the viability of fibroblasts decreased with the increase in quercetin concentration (*p* < 0.05, Figure 3(a)). The IC50 of quercetin treatment at 24 h was 20 μM. Fibroblasts were continuously treated with 20 μM quercetin, and their cell viability was detected at different times. The result showed that the viability of fibroblasts was found to decrease with time in a time-dependent manner (*p* < 0.05, Figure 3(b)). Edu staining showed that the number of positive cells decreased significantly following 20 μM quercetin treatment (p < 0.05, Figure 3(c)), indicating that quercetin had a significant inhibition on cell proliferation, while Western blot analysis yielded similar results. The expression of PCNA and Cyclin D1 decreased (*p* < 0.05, Figure 3(d)), which further demonstrated the inhibitory effect of quercetin on the proliferation of fibroblasts.

**Figure 4.**
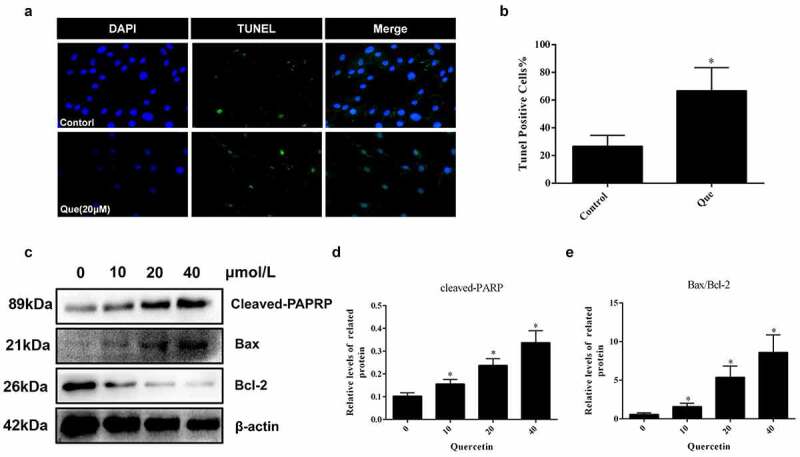
Quercetin induced fibroblast apoptosis in vitro. (a, b) Fibroblast apoptosis was analyzed by TUNEL staining for fibroblasts cultured with quercetin *p < 0.05 versus the control group. (c-e) The proteins levels of cleaved-PARP and Bax treated with different concentrations of quercetin. β-actin was used as a control.

In order to investigate the specific effect of quercetin on fibroblast apoptosis, fibroblasts with 20 μM quercetin were stimulated for 24 h. Afterward, TUNEL staining was used to detect whether quercetin had an effect on fibroblast apoptosis (*p* < 0.05, Figure 4(a-b)). Next, Western blot assay was performed to detect the apoptotic proteins, which demonstrated the promotional effect of quercetin on the fibroblast apoptosis. (*p* < 0.05, Figure 4(c-e)).

**Figure 5.**
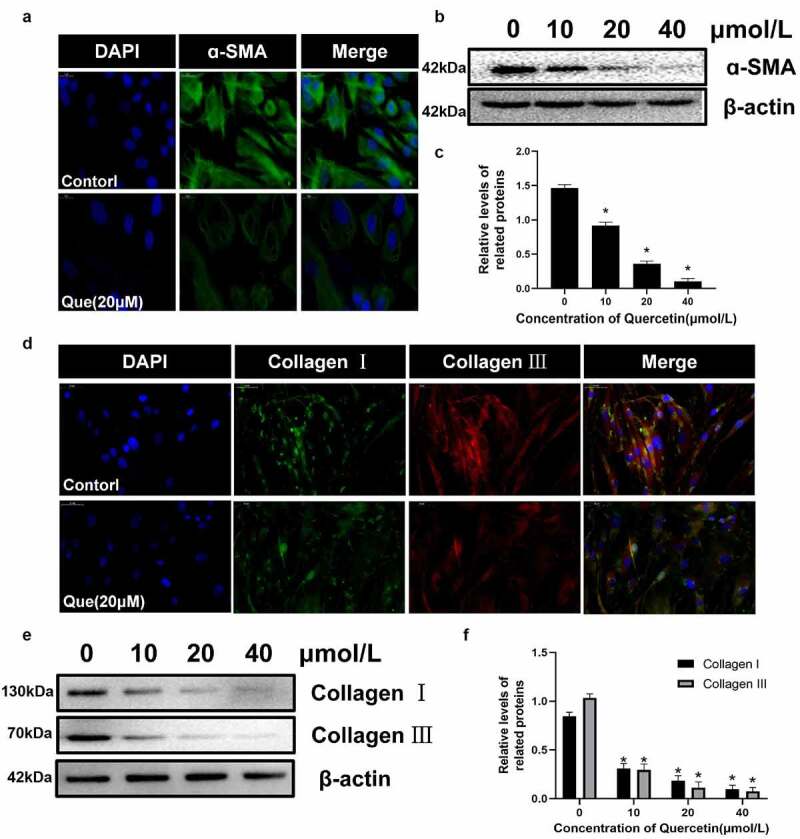
The influence of quercetin on fibroblasts differentiation and the expression of extracellular matrix .(a)Immunofluorescence staining for migration protein α – SMA treated with 20 µmol/L quercetin. (b, c) Under the intervention of different concentrations of quercetin, the protein levels of α-SMA in fibroblasts decreased in a concentration dependent manner. (d) Immunofluorescence staining for collagen I and collagen III. (e, f) The proteins levels of collagen I and III decreased in a concentration dependent manner treated with quercetin. . *Compared with the control group (*p* < 0.05).

The differentiation of myofibroblasts has been proved to be an important part of fibrosis, In addition, α – SMA is currently considered to be a marker of myofibroblasts. Therefore, immunofluorescence staining and western blot were used to detect the expression of migration protein α – SMA. We found that quercetin significantly reduced the expression of α – SMA (p < 0.05, Figure 5(a-c)). Collagen I and collagen III are considered to be the two main types of collagen in the extracellular matrix. The expression of collagen I and collagen III in fibroblasts was detected using immunofluorescence and western blot analysis. The results showed that quercetin could inhibit the formation of type I and III collagen in fibroblasts (*p* < 0.05, Figure 5(d-f)). All of the corresponding results indicated that quercetin can inhibit the proliferation, migration and collagen secretion of fibroblasts while promoting their apoptosis.

### Quercetin regulates fibroblast apoptosis and proliferation through autophagy, and mTOR mediated signaling pathway is involved

**Figure 6.**
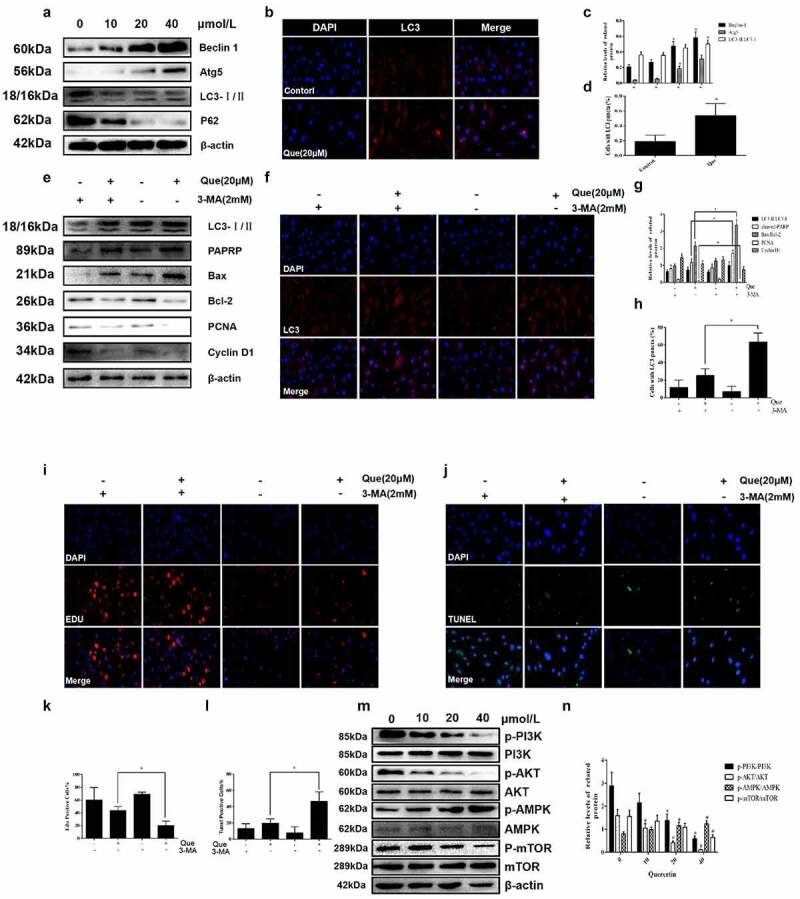
Quercetin regulates fibroblast apoptosis and proliferation through autophagy, and mTOR mediated signaling pathway is involved. (a, c)The expression levels of autophagy-related proteins Beclin-1, P62, LC3 and Atg5 in fibroblasts were analyzed by western blotting after cells were treated with various concentrations of quercetin for 24 h. (b, d) After 20 µmol/L quercetin treatment of fibroblasts for 24 h, immunofluorescence staining for flake LC3 fluorescent light. (e, f) After treated with Autophagy inhibitors 3-MA, the low expression levels of Cyclin D1 and PCNA were partly reversed, while the high expression levels of cleaved-PARP and LC3-II/LC3-I were partly inhibited. (g, h) Fibroblasts were exposed to 20 µmol/L quercetin or 2 mmol/L 3-MA, or a combined treatment of quercetin and 3-MA for 24 h. immunofluorescence staining for LC3 in fibroblasts . (i, j) Fibroblasts were exposed to 20 µmol/L quercetin or 2 mmol/L 3-MA, or a combined treatment of quercetin and 3-MA for 24 h. EdU staining showed that after treated with Autophagy inhibitors 3-MA, the proliferation limitation of fibroblasts induced by quercetin was reversed to a certain extent. (k, l) TUNEL staining for fibroblasts treated with 20 µmol/L quercetin and autophagy inhibitors 3-MA *p < 0.05 versus the control group. (m, n) The proteins levels of p-PI3K, p-AKT, p-MAPK and p-mTOR decreased in a concentration dependent manner treated with quercetin .β-actin was used as a control.

In order to study the specific mechanism of quercetin on the proliferation and apoptosis of fibroblasts, further experiments were performed. First, the expression of autophagy related proteins following quercetin were detected at different concentrations (0, 10 μM,20 μM,40 μM). With the increase in drug concentration, the results illustrated that the expression of Beclin 1, Atg5, and LC3 II/LC3 I increased, while that of p62 decreased (*p* < 0.05, Figure 6(a-c)). This trend was closely related to drug concentration, which exhibited a concentration dependent manner. Moreover, LC3 immunofluorescence images demonstrated the presence of a significantly increased number of LC3 dots in fibroblasts following treatment of 20 μM quercetin (*p* < 0.05, Figure 6(b-d)). To further explore the role of autophagy, autophagy inhibitor 3-MA was used. However, following the stimulation of 2 mM 3-MA, the low expression levels of Cyclin D1 and PCNA were partly reversed after 20 μM quercetin induction. Additionally, the high expression levels of cleaved-PARP and LC3-II/LC3-I were found to be partially inhibited (*p* < 0.05, Figure 6(e-g)). Meanwhile, the results of TUNEL, EdU assay and LC3 immunofluorescence yielded the same results (*p* < 0.05, Figure 6(f,h,i,j,k)), which indicated that autophagy was inhibited after application of 3-MA.

Finally, proteins related to the classical pathway of autophagy were then detected. To further investigate its mechanism, the expression levels of related proteins in the mTOR mediated signaling pathway were detected. As the concentration of quercetin rose, the expression levels of p-PI3K, p-mTOR, and p-AKT decreased, while that of p-AMPK increased in a concentration-dependent manner (*p* < 0.05, Figure 6(m,n)). The above results demonstrated that quercetin-induced negative regulation of mTOR mediated signaling pathway played an important role in the action of quercetin.

## Discussion

Surgery-induced extensive epidural fibrosis following laminectomy often results in negative effects on outcome and even causes failed back surgery syndrome (FBSS) [1,2]. Meanwhile, fibroblast proliferation plays an important role in the formation of EF [28]. Following surgical laminectomy, fibroblasts in the bone defect site are activated by inflammatory cytokines and growth factors to produce collagen and extracellular matrix. This activation produces excessive fibrous connective tissue to repair the local defects, eventually leading to epidural fibrosis [29].

Quercetin, a kind of major flavonoid found in many traditional Chinese medicines, is an effective substance for treatment [30]. It had been reported that quercetin could reduce the expression of extracellular matrix proteins and exert inhibitory effect on cell proliferation [31]. Due to its particularity, quercetin has recently been used to treat proliferative fibrosis and achieved satisfactory results [32,33].

In this study, the preventive effect of quercetin on epidural fibrosis scar adhesion was studied. The results showed that quercetin can effectively prevent the formation of EF, exhibiting a concentration dependent manner. The results of HE and Masson staining showed that quercetin could reduce the number of fibroblasts and collagen content in the operation area. Here, fibroblasts acted as the main effector cells of scar formation, while the amount of collagen determined the degree of fibrosis. These results fully explain that quercetin can reduce the number of fibroblasts and collagen content in the operation area to inhibit fibrotic scar adhesion in rats following epidural surgery, which is concentration dependent. The mechanism of quercetin that produces this effect is not clear. It is reported that quercetin can exert anti-tumor effects by inhibiting cell proliferation, promoting apoptosis and affecting autophagy [34]. Previous studies indicated that autophagy could downregulate mTOR expression to inhibit cell proliferation [15,16]. Hence, immunohistochemical staining was carried out to detect the expression of autophagy associated protein LC3 and p-mTOR. The results showed that with a rise in drug concentration, LC3 expression increased significantly while that of p-mTOR decreased. LC3 is the characteristic protein of autophagy; therefore, we can speculate that autophagy plays an important role in the prevention of epidural fibrosis scar adhesion by quercetin.

Autophagy is an important biological behavior of organisms, which is extremely conservative in evolution [12]. The regulation of autophagy is a very complex process, and existing research is incomplete. The regulation of autophagy can be divided into mTOR dependent and mTOR independent regulation. mTOR is a mammalian target of rapamycin [35]. As the most important protein in autophagy regulation, mTOR can sense multiple signals and regulate autophagy negatively [36]. mTOR plays an important role in cell biological processes, such as proliferation and apoptosis. mTOR is involved in many developmental processes, such as nerve regeneration and T lymphocyte activation [37]. In general, mTOR inhibits the activity of autophagy initiation molecule ATG 1, controlling the occurrence of autophagy [38]. Activated mTOR participates in many cellular functions by phosphorylating certain factors (4E-BP1 and P70S6K) in protein translation [39]. When a tumor occurs, its expression will be upregulated, and autophagy will be inhibited [40]. Most autophagy signal transduction pathways are conducted via the mTOR pathway; hence, as an important inhibitory pathway, it plays an irreplaceable role in autophagy [41].

In the cytological experiments, CCK-8 showed that quercetin could effectively inhibit the viability of fibroblasts in a concentration and time-dependent manner. Afterward, Edu and TUNEL staining demonstrated that quercetin could inhibit the proliferation of fibroblasts and increase its apoptosis. Western blot analysis of proliferation and apoptosis related proteins yielded similar results. Subsequently, the expression of migration associated protein α – SMA, collagen type 1 and collagen type 3 were detected. The results illustrated that quercetin could reduce the expression of α – SMA, type 1 collagen and type 3 collagen and that quercetin has a regulatory effect on the biological behavior of fibroblasts. Quercetin can inhibit the viability and reduce the number of fibroblasts through the regulation of proliferation, apoptosis, and migration.

In order to further clarify the role of autophagy in the process of quercetin, LC3 immunofluorescence detection was done, which found that the number of fluorescent spots increased significantly in the drug group, indicating that autophagy was activated after quercetin. The autophagy related proteins beclin-1, LC3, ATG5 and p62 were then detected by Western blot. Accordingly, beclin-1, LC3-II/I ratio and ATG5 expression increased with a rise in quercetin concentration, while p62 decreased in a concentration-dependent manner, further suggesting the occurrence of autophagy. Quercetin can induce autophagy in fibroblasts. In order to further explore the relationship between autophagy and the effects of inhibiting proliferation and inducing apoptosis, autophagy inhibitor 3-MA was used. The cells were divided into groups, pretreated with 3-MA, intervened by quercetin, and detected by LC3 immunofluorescence staining. The results showed that the number of fluorescent spots in fibroblasts pretreated with 3-MA was lower than that in the control group after quercetin intervention. The expression of LC3 protein was then detected by Western blot. The results also showed that the LC3-II/I ratio in fibroblasts pretreated with 3-MA was lower than that in the control group. These results fully indicate that 3-MA can effectively inhibit quercetin induced autophagy in fibroblasts. Following 3-MA pretreatment of fibroblasts, Western blot was used to detect the proliferation and apoptosis of the related proteins. As autophagy in fibroblasts was inhibited, quercetin’s effect on cell viability and proliferation was found to be effectively reduced, while its ability to induce apoptosis of fibroblasts was partially reversed. Finally, the protein expression of the key upstream pathways of mTOR were detected, which illustrated that the expression levels of p-PI3K and p-AKT decreased while p-AMPK increased, indicating that the PI3K/Akt and AMPK/mTOR pathways were inhibited, thus inhibiting the activity of mTOR.

The clear mechanism of adhesion formation is very complex. In this study, we studied the effect of quercetin on fibroblast proliferation and reduction of intra-articular fibrotic scar adhesion. In our study, after laminectomy, no rats had any side effects, such as surgical incision infection, epidermal necrosis or death. However, the systemic complications of quercetin intragastric administration are unclear. In addition, the clinical significance of quercetin treatment in patients has not been answered, and more research is needed in the future.

## Conclusions

The results above demonstrated that quercetin could effectively prevent the occurrence of epidural fibrosis scar adhesion, which may occur through the mTOR mediated signaling pathway in order to regulate the proliferation, apoptosis, migration and other biological behaviors of fibroblasts. Although this study is still relatively basic with various limitations, it provides novel insight and research directions for the clinical prevention of epidural fibrosis scar adhesion.

## Data Availability

All data generated or analyzed during this study are included in this published article.

## Highlights

• Quercetin induces autophagy in the surgical area;

• Quercetin affects the biological behaviors of fibroblasts such as apoptosis, proliferation and migration;

• Quercetin regulates fibroblast apoptosis and proliferation through autophagy, and mTOR mediated signaling pathway is involved.

## Abbreviations

3-MA: 3-Methyladenine
CCK-8: cell counting kit-8
HE: hematoxylineosin
PARP: poly ADP-ribose polymerase
DMEM: Dulbecco’s Modified Eagle’s medium
FBS: fetal bovine serum
PBS: phosphate buffer saline
RIPA: radioimmune-deposition
SDS-PAGE: sodium dodecyl sulfate-polyacrylamide gel electrophoresis
PVDF: polyvinylidene difluoride
TBST: tris-buffered saline and Tween20
